# Ultrasound-Guided External Oblique Intercostal Block as Part of Multimodal Analgesia for Unplanned Open Cholecystectomy

**DOI:** 10.7759/cureus.61606

**Published:** 2024-06-03

**Authors:** Malcolm Lee, Michael Ayad, Jose L Diz Ferre, Lori Ann Oliver, Nicholas Swerchowsky, Sabry Ayad

**Affiliations:** 1 Outcomes Research Consortium, Ohio University Heritage College of Osteopathic Medicine, Cleveland, USA; 2 Outcomes Research Consortium, Lake Erie College of Osteopathic Medicine, Cleveland, USA; 3 Outcomes Research, Cleveland Clinic, Cleveland, USA; 4 Anesthesiology, Cleveland Clinic Fairview Hospital, Cleveland, USA; 5 Anesthesiology, Cleveland Clinic, Cleveland, USA

**Keywords:** continuous peripheral nerve block, ultrasound-guided, lap converted to open cholecystectomy, multi-modality pain management, external oblique nerve block

## Abstract

We present the case of a 56-year-old female with a significant medical history of cholelithiasis and recurrent choledocholithiasis. Following an elective cholecystectomy, an obstructing gallstone in the common bile duct led to a series of interventions, including endoscopic retrograde cholangiopancreatography and stent placement. The patient was scheduled for a robot-assisted laparoscopic common bile duct exploration. Due to severe adhesions, the procedure was converted to open with a large right upper quadrant incision. Intraoperative continuous external oblique block and catheter placement were performed at the end of surgery in the OR. Peripheral nerve blocks have become an integral part of multimodal pain management strategies. This case report describes the successful implementation of an ultrasound-guided right external oblique intercostal block and catheter placement for postoperative pain control and minimization of opioids. This case highlights the efficacy and safety of ultrasound-guided peripheral nerve blocks for postoperative pain management. Successful pain control contributed to the patient's overall postoperative recovery.

## Introduction

Upper abdominal incisions such as oblique subcostal laparotomies are associated with significant pain and respiratory compromise and are often managed with either epidural or peripheral nerve blocks. These procedures can be technically difficult to perform due to several factors including the patient's body habitus (e.g. obesity) and proximity to the surgical site as well as the skill of the regional anesthesiologist. External oblique intercostal (EOI) block is a novel regional procedure that is simple, relatively easy to execute and has been associated with excellent pain control [1]. Our case highlights the use of continuous EOI blocks for postoperative pain management in a robot-assisted laparoscopic converted to open common bile exploration.

## Case presentation

We report here the case of a 56-year-old female, BMI 33.25 (93.4 kg) with a past medical history significant for cholelithiasis and recurrent choledocholithiasis. She underwent elective cholecystectomy with residual obstructing gallstone in the common bile duct that led to a series of interventions, including endoscopic retrograde cholangiopancreatography and stent placement, which were unsuccessful.

Surgery was recommended after she presented to the general surgery office complaining of sharp epigastric pain that radiates to her back without food association. Considering her medical history, the patient was later scheduled for robot-assisted laparoscopic common bile duct exploration. Due to significant adhesions, the procedure was converted to open with a large right upper quadrant subcostal incision. Placement of an epidural catheter was no longer possible, so a decision was made to proceed with the placement of EOI catheters at the end of the surgery prior to extubation. A choledocoduodenostomy was performed. While in the operating room, the patient had a right EOI peripheral nerve block placed for postoperative pain management [2,3]. 

**Figure 1:**
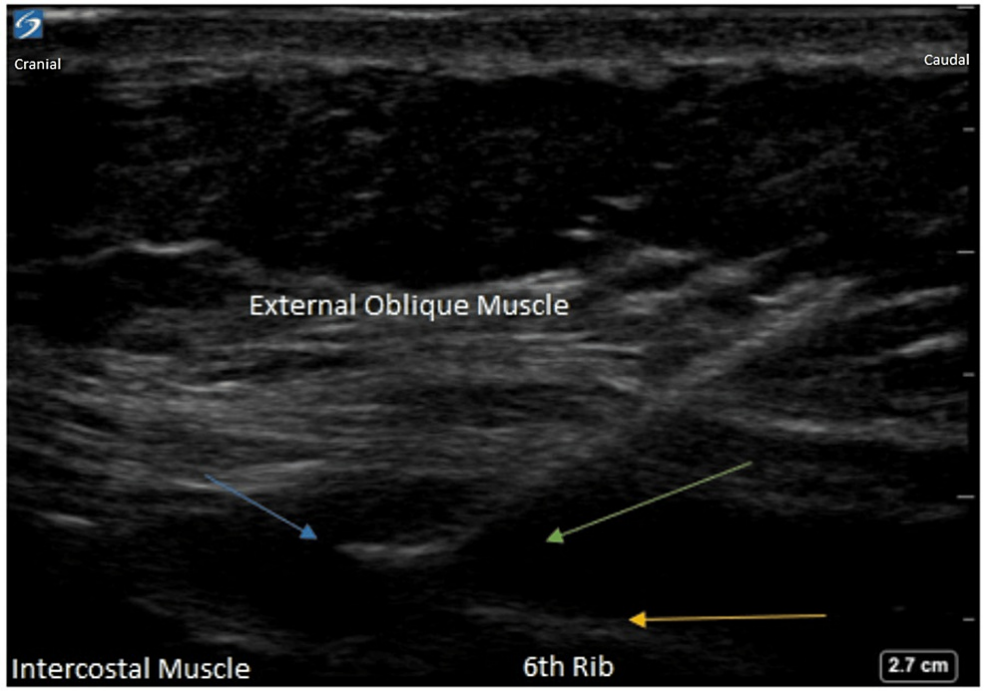
Ultrasound-guided external oblique intercostal block. Green arrow: injected solution; blue arrow: tip of the needle; yellow arrow: sixth rib.

These blocks were performed in the supine position using both anatomical landmarks and ultrasound imaging for confirmation (see Figure 1). A high-frequency linear 13-6 MHz ultrasound probe was placed on the chest wall medial to the mid-axillary line over the sixth rib and parasagittal in orientation.​ An 18G echogenic needle was then advanced from cephalad to caudad deep to the EOI muscle using hydro-dissection with saline. The needle was then advanced 1-2 cm and the catheter was subsequently placed an additional 4 cm beyond the needle tip under ultrasound guidance and secured. A total of 30 ml of 0.5% bupivacaine was administered. Confirmation of the catheter's correct placement was achieved through ultrasound visualization of the dispersion of the local anesthetic in the targeted area. 

The patient was maintained on ropivacaine 0.2% infusion through the catheter and placed on a multimodal regimen including demand only hydromorphone patient-controlled analgesia and acetaminophen 1 g every 6 hours. She was later transitioned to oxycodone 5-10 mg by mouth (PO) every 4 hours PRN on postoperative day (POD) 2. The patient did not experience nausea or vomiting and reported having a pain score of 0/10 at rest and 2/10 with ambulation on POD3. EOI catheters were removed on POD4 with good pain control.​ 

## Discussion

Epidural analgesia is widely regarded as the gold standard for postoperative pain management after open abdominal procedures due to its efficacy in providing analgesia [4]. Its placement takes place usually before induction of general anesthesia, typically with the patient in the sitting or lateral position. Therefore, it can be challenging to perform postoperatively due to patient discomfort and limited mobility. This limitation has led to an increased interest in fascial plane blocks such as the transversus abdominis plane (TAP) and quadratus lumborum (QL) blocks, which can be administered with the patient in the supine position.

The TAP block involves the injection of local anesthetic into the plane between the internal oblique and transversus abdominis muscles, providing analgesia for the T10-12 dermatomes and it is particularly effective for lower abdominal incisions [5]. Subcostal TAP blocks, while useful for vertical incisions, may not provide adequate coverage for oblique upper quadrant incisions due to the lack of blockade of the lateral cutaneous branches of the upper abdominal intercostal nerves. On the other hand, the QL blocks target the plane between the QL and psoas major muscles, offering analgesia for the T7-L1 dermatomes, which is also suitable for a variety of abdominal incisions [6].​ 

However, EOI is a novel fascial plane block described by Elsharkaway in 2021 that provides analgesia to both the anterior and lateral abdominal walls (T6-10) [2]. An advantage of this block is its reasonably fast learning curve and execution due to the superficial nature of the block, which is an advantage for placement in obese patients. This regional technique offers effective pain relief and can be performed in the supine position, making it a valuable alternative to epidural analgesia in the postoperative period. In our case, the patient achieved optimal pain control immediately after surgery. She recovered bowel function without nausea or vomiting in three days and was able to be discharged on day 4.

## Conclusions

In summary, while epidural analgesia remains the gold standard for postoperative pain management for open abdominal procedures, fascial plane blocks such as the TAP, QL, and EOI blocks offer promising alternatives, especially in cases where epidural placement is not feasible and/or contraindicated.

The use of ultrasonography has resulted in the creation of novel fascial plane blocks that have helped to improve patients' postoperative pain and overall recovery. This case highlights the efficacy and safety of ultrasound-guided continuous EOI nerve blocks for postoperative pain management as a rescue block. It is relatively easy to perform in obese patients for procedures with oblique incisions such as in our case.
